# More Than Talking About the Weekend: Content of Case-Irrelevant Communication Within the OR Team

**DOI:** 10.1007/s00268-017-4442-4

**Published:** 2018-01-09

**Authors:** Lukas W. Widmer, Sandra Keller, Franziska Tschan, Norbert K. Semmer, Eliane Holzer, Daniel Candinas, Guido Beldi

**Affiliations:** 1 Department of Visceral Surgery and Medicine, University Hospital of Bern, 3010 Bern, Switzerland; 2 University of Neuchâtel, Institute of Work and Organizational Psychology, Neuchâtel, Switzerland; 3 Department of Psychology, University of Berne, Bern, Switzerland

## Abstract

**Background:**

Case-irrelevant communication (CIC) is defined as “any conversation” irrelevant to the case. It includes small talk, but also communication related to other work issues besides the actual task. CIC during surgeries is generally seen as distracting, despite a lack of knowledge about the content of CIC and its regulation in terms of adjustments to the situation of CIC. Primary goal of the study was to evaluate CIC content; secondary goal was to evaluate whether surgical teams regulate CIC according to different concentration demands of surgical procedures.

**Methods:**

In 125 surgeries, 1396 CIC events were observed. CIC were content coded into work-related CIC (pertaining to other tasks or work in general) and social CIC (pertaining to acquaintance talk, gossip, or private conversation). The impact of different phases and the difficulty of the surgical procedure on CIC were assessed.

**Results:**

Work-related CIC were significantly more frequent (2.49 per hour, SD = 2.17) than social CIC (1.42 per hour, SD = 2.17). Across phases, frequency of work-related CIC was constant, whereas social CIC increased significantly across phases. In surgeries assessed as highly difficult by the surgeons, social CIC were observed at a lower frequency, and less work-related CIC were observed during the main phase compared to surgeries assessed as less difficult.

**Conclusion:**

The high proportion of work-related CIC indicates that surgical teams deal with other tasks during surgeries. Surgical teams adapt CIC according to the demands of the procedure. Hospital policies should support these adaptations rather than attempt to suppress CIC entirely.

## Introduction

Performing surgery is a complex task that requires high concentration. However, interruptions and distractions that may threaten this concentration are frequently observed during surgeries [1–4]. A potential distractor is case-irrelevant communication, which is the focus of this study. In particular, this study aims to describe (1) type and frequency of case-irrelevant communication and (2) the regulation of case-irrelevant communication within the surgical team.

Communication within a surgical team during the procedure can be related to the actual case (case-relevant communication) or it can be case-irrelevant (CIC). CIC is defined very generally as “any conversation” irrelevant to the case and may include small talk, but also communication related to other work issues besides the actual task (e.g., discussions about other patients; scheduling of other procedures) [5, 6].

Because CIC is not necessary or useful for the task at hand, it is often seen as a “communication problem” that needs to be dealt with in the operating room, and is studied together with other distractors [7]. Compared to other distractor categories such as door openings or noise events, CIC is more frequently observed during the intraoperative or early postoperative phases [2, 3, 6, 8–14]. Frequencies of CIC range from about every 20 min in shorter (<4 h) [12] to every 10 min in long open abdominal procedures [15].

Because the surgical team is involved in generating CIC, it potentially binds more attention of the surgical team than other distractors. Thus, CIC could be particularly harmful for concentration [3, 12]. Although surgeons report less concentration if more CICs are observed, recent reports show that the distracting potential of CIC is in the medium range and distracts less than issues involving technical equipment or procedural problems [8, 13]. A recent study suggests that the distracting potential of overall CIC is highly dependent on the context within the procedure, as CIC impacts on clinical outcome only when frequent during the closing phase of the surgery [15].

Despite its potential to distract, CIC may exhibit important other, even positive, functions. First, CIC related to other aspects of work may be required to solve other problems that typically occur simultaneously to surgeries in clinical practice, such as responding to questions about other patients, or organizational issues [16]. Indeed, 25% of observed CIC have been found to be related to other patients [6]. A second important function of CIC may be social. Small talk can relax the atmosphere within the surgical team and release tension and thereby be important for good teamwork [17–19]. Thus, CIC may contribute to a good social climate and may be a sign of transformational leadership, a form of leadership which is advantageous in the OR [20].

Regulation of CIC within the surgical team is likely to be highly complex. Most of CIC is initiated by surgeons [2, 6, 13], and it is almost always targeted at other surgeons [6]. CIC can in general be controlled by the surgical team, e.g., by avoiding CIC when the concentration demands of the tasks are high [9, 21]. This type of regulation is analogous to talking to a passenger while driving: Although the distracting potential of conversations with passengers has been shown, drivers as well as passengers react to changes in driving conditions by limiting their conversations in heavy traffic [22]. It is thus reasonable to expect surgeons to engage less in CIC in phases of the procedure when high concentration is needed; as has been observed for other distractors [23]. The middle phase of a surgical procedure has been shown to be associated with the highest difficulty, whereas early or late phases (opening and closure) typically are less challenging [12, 13, 24]. One can thus expect that surgical teams regulate CIC specifically in the middle or very difficult phases of a surgical procedure.

In sum, CIC during surgery may be necessary, helpful or distracting. However, neither the content CIC nor the regulation of CIC within the surgical team has been explored in detail. Therefore, the primary goal of the current study is to explore the content of CIC during elective surgical procedures, and the secondary goal is to investigate the regulation of CIC within the surgical team across different phases of surgical procedures of different complexity.

## Materials and methods

**Fig. 1:**
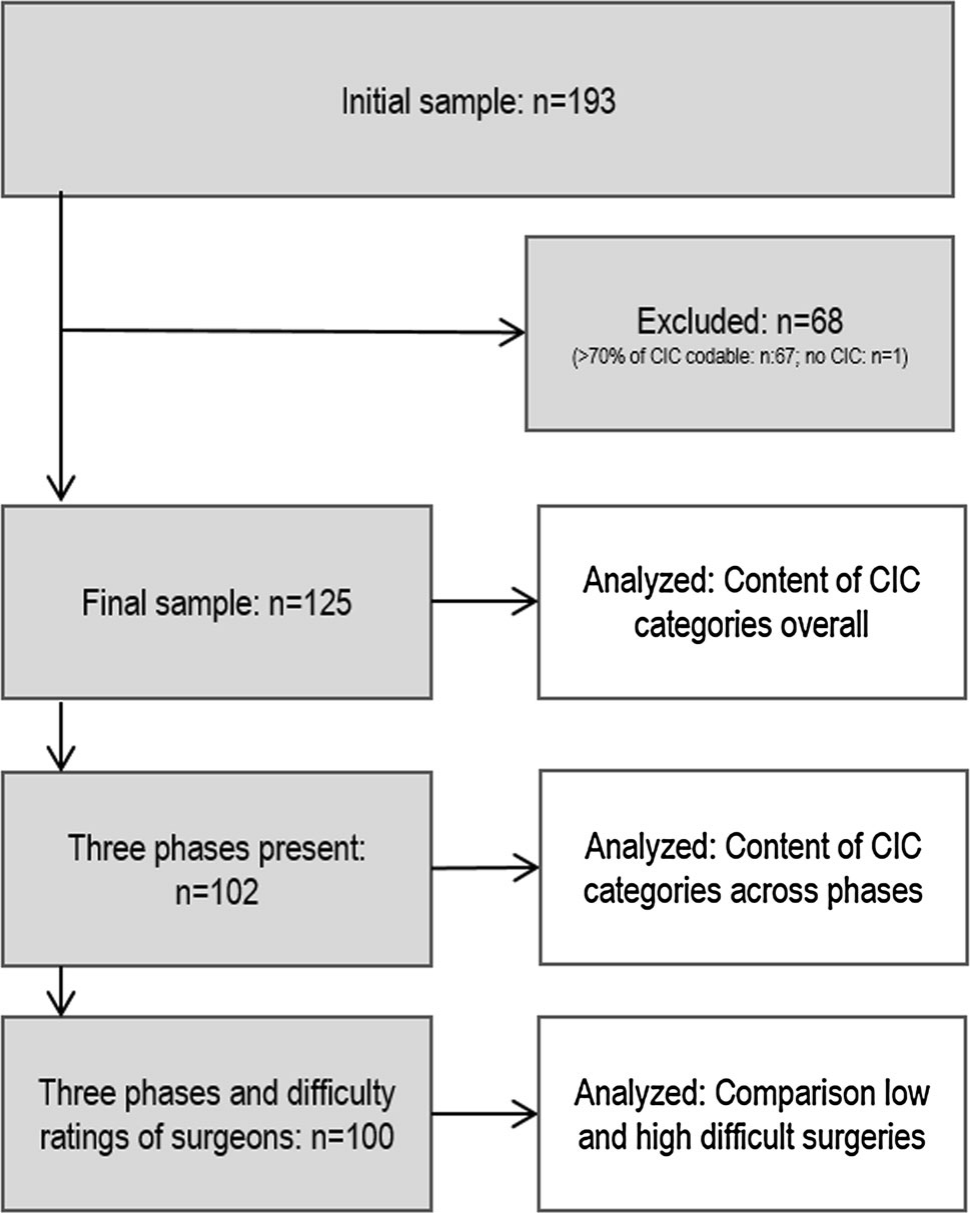
Flowchart

Inclusion criteria for observations were open abdominal procedures with an expected duration of at least 1 h and the availability of observers. A total of 193 procedures were observed in a European University hospital. In one surgery, no CIC was observed. Sixty-seven surgeries had to be excluded because the observers could not determine CIC content precisely enough (e.g., because team members talked at a very low voice) for more than 70% of the CIC. The final sample consists of 125 procedures (Fig. 1), performed by 20 different main surgeons.

The internal institutional review board agreed to the observation of the surgical teams. Individuals were consented with an opt-out procedure, as each member of the team could at any moment ask the observational team to leave.

### Observation and content coding of CIC

Each surgery was observed by trained observers (work and organizational psychologists), using a validated event-based observational system [25]. The observation period was between skin incision and end of skin closure. The observers were seated in about 1.5 m distance from the operating table, opposite to the lead surgeon. The observers coded each verbal exchange within the sterile team and between at least one member of the sterile team and the anesthesiologists. CIC was coded if the surgical team engaged in topics that were not related to the patient or the procedure.

If the team engaged in a CIC, the observers first noted that the CIC took place; the time was automatically recorded. If the observers could understand the content of CIC, they summarized it in the comment section of the coding application. Each observational comment was then content coded [26] into two main categories (related to work vs. small talk) with three distinct sub-categories each, according to the following description.

Main category work-related CIC:

1. *Other tasks or patients* Examples are a conversation about an assistant physician who was asked to help out in a surgery in another OR, or a conversation about the next patient or a patient in the emergency room.
2. *Work and medicine in general* Examples are a conversation about reducing the number of instruments that are required during operations; the surgeons discussing how to avoid back problems while doing surgery.
3. *Context talk related to the surgery* included comments about the context of the current surgery or its organizational aspects. Examples are the general quality of technical devices; the student asking for permission to leave and explaining the reasons.

Main category social CIC (small talk):

4. *Acquaintance talk* included introducing new collaborators and talking about one’s own biography. Examples are that the surgeon asks the student to repeat her name and asks how long she will stay in the service; a surgeon talks about his work biography.
5. *Gossip* includes exchanging information about other people. Examples are talking about opinions of a colleague not present, talk about hospital policies.
6. *Private conversations* include talking about one’s own personal life (excluding professional biography). Examples include talking about one’s children or pets; talking about a recent popular vote.

If a conversation involved several categories, the most predominant category was coded, so that each CIC represents only one category. CICs that could not be categorized were noted. For validation purposes, two coders independently categorized 22% of the comments. Interobserver agreement (Cohen’s weighted kappa) was 0.76, which indicates good interobserver reliability: the rest of the comments were coded by the first author [27].

### Case-related communication

Case-related communication was coded if the surgical team engaged in topics related to the patient or the procedure, including case-related teaching and leadership [25].

### Difficulty of surgery

After each surgery, just before leaving the operation room, the surgeons completed a short standardized questionnaire to evaluate the difficulty of the operation. Difficulty was assessed with the question “How difficult was the surgery for you?” and assessed on a 7-point Likert type scale with scores between 1 (very easy) to 7 (very difficult). If more than one surgeon was present, their difficulty assessments were averaged. Difficulty levels were split at the mean (4.5) in low and high difficult procedures; thus, 49.6% of the surgeries were categorized as low difficulty. Questionnaires were confidential.

### Phase of surgery

Three different phases of the surgery were distinguished according to the presence of the main and most experienced (senior) surgeon [28, 29]. In 102 of the 125 surgeries, the senior surgeon joined the team after the preparatory phase, stayed for the main phase, and left the surgery before the closing phase, this is customary in this institution, where fellows with board examination often are responsible for the first and last part of the procedure. The main phase can be considered the most difficult part of the surgery [29]. All surgical steps during this period were either performed or were closely supervised by the senior surgeon. Thus, phases were defined as follows:

- phase 1: before the senior surgeon is present
- phase 2: senior surgeon present
- phase 3: senior surgeon left the operation

### Outcome parameters

The primary outcome of the study was the frequency of content of CIC, according to the main and sub-categories. The secondary outcome was the content of CIC of the two main categories for easy and difficult surgeries across the three phases.

### Statistical analyses

For statistical analysis, we used SPSS (IBM Corp. Released 2013. IBM SPSS Statistics for Macintosh, Version 24.0. Armonk, NY: IBM Corp.). Non-parametrical data are displayed as median and interquartile range (IQR), parametrical data as mean and standard deviation (SD). Inter-rater agreement was assessed using Cohen’s weighted Kappa statistics. A *P* value below 0.05 was defined as statistical significant. Mann–Whitney *U* test were used for comparisons, *t*-tests for repeated measures and analyses of variances for repeated measures were used to compare CIC across and phases. Post hoc comparisons were Bonferroni corrected.

## Results

### Frequency of CIC

**Fig. 2:**
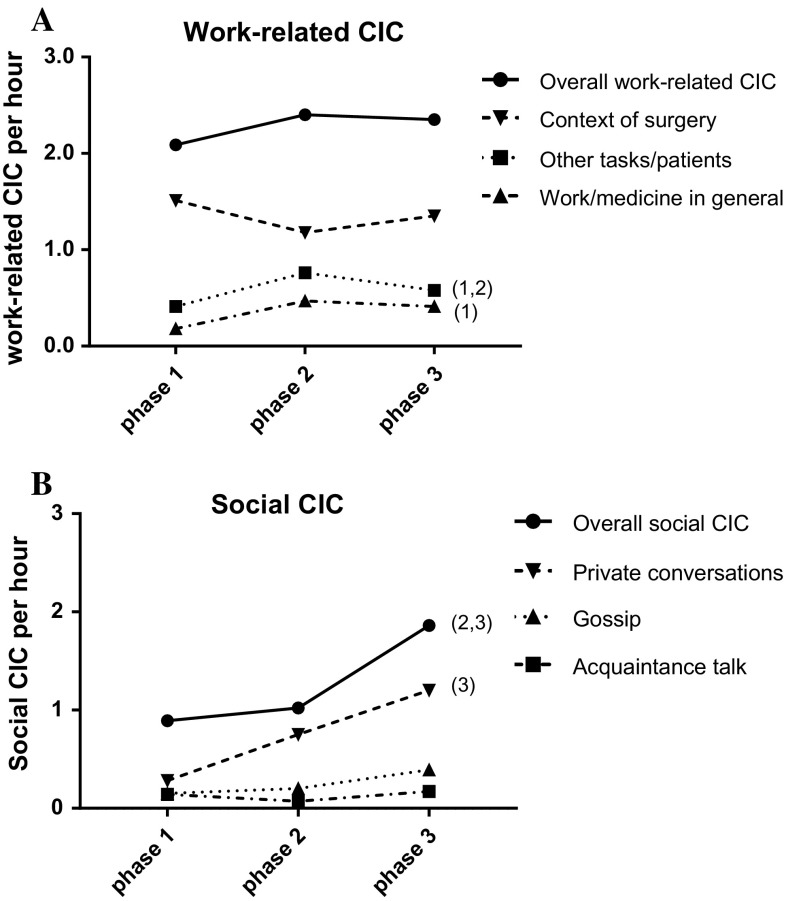
**a** Frequency of work-related CIC across phases: (1) significant difference between phase 1 and phase 2; (2) significant difference between phase 2 and phase 3. **b** Frequency of social CIC across phases: (2) significant difference between phase 2 and phase 3; (3) significant difference between phase 1 and phase 3

In the 125 surgeries included (Table 1), 1396 CICs were observed; with a mean of 11.17 per surgery (SD = 8.79), a range of 1–48 per surgery, and a density of 2.97 CIC (SD = 3.50) per hour of surgery. Work-related CICs were observed at a frequency of 2.49 observations per hour with a standard deviation (SD) of 2.17, social CIC were observed at 1.42 (SD 2.17) per hour (*P* < 0.001). During procedures, the frequency of overall work-related CIC did not change significantly; however, the frequency of social CIC was significantly higher in the last phase (Table 2 and Fig. 2a, b). CIC amounted to 12.89% (SD = 10.13%) of all observed communication within the sterile team.

**Table 1:** Operative procedures and descriptive statistics

	(*n* = 125)
Patient age (SD)	61.5 (14.8)
Duration of surgery in hours (SD)	4.5 (2.0)
Patient gender (% males)	68 (55.9%)
Type of surgery	
Hepatobiliary/pancreatic	63 (50.4%)
Upper GI tract	24 (19.2%)
Lower GI tract	22 (17.6%)
Other	16 (12.8%)
Average surgeon’s evaluation of difficulty level (range 1–7, SD)	4.48 (1.05)
Proportion CIC content coded (SD)	88.9% (10.4)

*SD* standard deviation

**Table 2:** Content categories of CIC overall, and in phase 1, 2 or 3, respectively

	Overall mean (SD)/per hour	min–max/per hour	Phase 1 mean (SD)/per hour	Phase 2 mean (SD)/per hour	Phase 3 mean (SD)/per hour	*P* value (phases)
	*N* = 125		*n* = 102			
Work-related CIC	2.49 (2.17)	0–14.7	2.09 (2.97)_a_	2.40 (2.29)_a_	2.35 (3.09)_a_	0.618
Other tasks/patients	0.70 (0.95)	0–8.3	0.41 (0.80)_a_	0.76 (0.86)_b_	0.58 (1.27)_a,b_	**0.028**
Work/medicine in general	0.44 (0.78)	0–6.0	0.18 (0.83)_a_	0.47 (1.01)_b_	0.41 (0.86)_c_	**0.029**
Context of surgery	1.34 (1.11)	0–5.4	1.51 (2.68)_a_	1.18 (1.32)_a_	1.35 (2.37)_a_	0.517
Social CIC (small talk)	1.42 (2.17)	0–20.2	0.89 (1.52)_a_	1.02 (1.25)_a_	1.86 (3.83)_b_	**0.005**
Acquaintance talk	0.13 (0.23)	0–1.0	0.14 (0.42)_a_	0.07 (0.18)_a_	0.17 (0.60)_a_	0.27
Gossip	0.26 (0.48)	0–2.4	0.15 (0.41)_a_	0.20 (0.43)_a_	0.39 (1.33)_a_	0.1
Private conversations	1.02 (2.01)	0–20.2	0.28 (1.17)_a_	0.75 (0.97)_a,b_	1.20 (3.45)_b_	**0.038**

Bold values indicate statistical significance (*P* < 0.05)

Phases with different subscripts were significantly different from each other (across rows, Bonferroni-corrected post hoc tests)

### Regulation of CIC

We tested whether the surgical teams regulated the frequency of CIC according to the difficulty of the procedure and the phase. The frequency of work-related CIC was not different for low and high difficult surgeries overall. However, in phase 2, significantly less work-related CIC was observed in difficult surgeries (Table 3). The frequency of social CIC was significantly lower in difficult surgeries than in less difficult surgeries. However, there was no statistically significant difference within the three phases of the surgery (Table 3).

**Table 3:** Work-related and social CIC across phases for surgeries with high and low difficulty ratings

	Difficulty level	Phase 1	Phase 2	Phase 3	Overall
		Before senior surgeon arrives	Senior surgeon present	After senior surgeon leaves	
		Median (IQR)	Median (IQR)	Median (IQR)	Median (IQR)
Work-related CIC	Low	1.42 (3.05)	2.22 (2.45)	1.42 (5.40)	2.37 (2.20)
	High	1.23 (3.08)	1.39 (1.80)	1.39 (3.03)	1.93 (1.54)
	*P* ^a^	0.634	**0.023**	0.854	0.080
Social CIC	Low	0 (1.51)	0.8 (1.93)	0.87 (2.69)	1.18 (1.76)
	High	0 (0.99)	0.61 (1.12)	0.68 (1.77)	0.73 (1.19)
	*P* ^a^	0.42	0.24	0.414	**0.023**

Bold values indicate statistical significance (*P* < 0.05)

*IQR* interquartile range

^a^*P*-values are based on M–W nonparametric tests

## Discussion

The study showed that CIC could be clearly distinguished in work-related CIC and social CIC. Overall, CIC did not occur very frequently, with about 2.5 work-related CIC and 1.4 social CIC per hour; only about 13 percent of all communication was CIC. Work-related CIC occurred more frequently, but overall, remained constant across procedures, whereas social CIC density significantly increased throughout a procedure. Within work-related CIC, conversations related to the context of the surgery were most prevalent.

The presence of the senior surgeon critically influenced the frequency of work CIC related to other tasks/patients and general topics, as these were more often observed in the main operating phase with the most senior surgeon present. This may be the consequence of different positions within the hierarchical structure: The most senior surgeon may more often address specific organisational questions than more junior surgeons. The potential negative, distracting aspect of work-related CIC may be attenuated, because during difficult surgeries, the surgical teams engaged in significantly less work-related CIC during the second, the main phase. This indicates that the teams regulated work-related CIC according to varying concentration requirements.

The frequency of social CIC in general was highest during the last phase of the surgery, after the senior surgeon had left. This increase is mainly due to private conversations. The increase may represent a more relaxed social climate after the most difficult main phase—although it cannot be excluded that the effect is simply due to the fact that the senior surgeon has left. As social CIC implies rather low concentration demands [30], it could also be that fatigue after long operations contributed to the increase of social CIC. In that case, CIC may represent a surrogate parameter for decreasing concentration of the team. Overall, but not across phases, the surgical team engages in less social CIC in difficult surgeries. This, again, shows that the surgical teams adapted to the higher concentration demands in difficult surgeries.

Overall, the results show that if surgical teams do not communicate about the patient or the surgery at hand, they more often engage in work-related CIC than in social CIC. This indicates that they are dealing with other work-related aspects during surgeries. Although work-related CIC may be a distractor for the surgery at hand, it may be functional for the other tasks surgeons have to do outside of the OR.

Social CIC may be good for social aspects, but questionable with regard to patient outcomes, as a previous study showed [15]. Again, surgical teams regulate social CIC if concentration demands are high. Given these and previous findings, we propose that social CIC need to be assessed specifically in future studies in order to identify any potential impact on concentration and quality, but also on patient outcomes.

The results of this study do not support a recommendation for changes in general policies in the operating room with regard to CIC [8, 31]. Both work and social CIC seem to be at least partially functional and should neither be avoided nor completely suppressed. Work CIC may be necessary for the coordination of work beyond the actual surgery and social CIC are may be good for group climate [12]. However, CIC should be regulated in accordance with the concentration demands of the situation.

As a conclusion, CIC is more diverse than simple small talk and should be distinguished in work-related and social CIC. Variations of CIC throughout the phases of surgery and according to the difficulty of the surgery indicate that the surgical teams adapt their CIC activity to the task at hand. Policies should support these natural adaptations rather than attempt to suppress CIC.
